# Randomised, Double Blind, Controlled Trial of the Provision of Information about the Benefits of Organ Donation during a Family Donation Conversation

**DOI:** 10.1371/journal.pone.0155778

**Published:** 2016-06-20

**Authors:** Steve John Philpot, Sarah Aranha, David V. Pilcher, Michael Bailey

**Affiliations:** 1 Alfred Health, Melbourne, Victoria, Australia; 2 DonateLife Victoria, Melbourne, Victoria, Australia; 3 Monash University, Melbourne, Victoria, Australia; 4 Department Epidemiology and Preventive Medicine, Monash University, Melbourne, Victoria, Australia; 5 Australian and New Zealand Intensive Care Research Centre, Melbourne, Victoria, Australia; Clínica Universidad de Navarra, SPAIN

## Abstract

**Introduction:**

It is unclear how much information should be provided to families of potential organ donors about the benefits of organ donation. Whilst this information is material to the donation decision, it may also be perceived as coercive.

**Methods:**

Randomised, double blind, controlled trial in which community members watched one of two videos of a simulated organ donation conversation that differed only in the amount of information provided about the benefits of donation. Participants then completed a questionnaire about the adequacy of the information provided and the degree to which they felt the doctor was trying to convince the family member to say yes to donation.

**Results:**

There was a wide variability in what participants considered was the “right” amount of information about organ donation. Those who watched the conversation that included information about the benefits of donation were more likely to feel that the information provided to the family was sufficient. They were more likely to report that the doctor was trying to convince the family member to say yes to donation, yet were no more likely to feel uncomfortable or to feel that the doctor was uncaring or cared more about transplant recipients than he did for the patient and their family.

**Conclusions:**

This study suggests that community members are comfortable with health care staff providing information to family members that may be influential in supporting them to give consent for donation.

## Introduction

In Australia, less than 1% of all deaths occur in circumstances that make organ donation possible[1]. There are more than 1,600 people currently awaiting organ transplantation in Australia[1]. It is therefore important that organ donation is maximised by identifying all potential donors and also by taking measures to increase the organ donation consent rate.

Recent surveys of Australian community members have shown that a large majority of people surveyed believe that organ donation and transplantation is a worthwhile process and that they would wish to be a donor if they were ever in a position to do so[2]. Yet, when asked to make a surrogate decision on behalf of a family member, only 58% of families of potential donors consent to organ donation[3]. This suggests that there are people who would support donation for whom donation is declined by family members making an incorrect surrogate decision. It is likely that this is, in part, because families are asked to make a decision about organ donation at a time of recent or impending bereavement, and the decision that they make is influenced by factors such as grief, fatigue, misunderstanding of medical information, and difficulty processing new information[4–8]. Families who decline organ donation are more likely to question their decision months to years later than families who consent to donation[9,10].

The information given by health care staff when discussing donation has an important influence upon consent rates[4,11–13]. For example, requests for donation which are felt to imply that the requestor themselves is not supportive of donation yield a low consent rate, as do conversations which do not include discussion about the benefits of donation for transplant recipients[4]. Families are more likely to donate if conversations include information about the rarity of the opportunity for donation[2].

Recent research has supported the presence of a trained requestor in the family donation conversation[14]. These trained requestors have undertaken specialised communications training specific to discussing organ donation, including which information should be given to families who are considering donation on behalf of a loved one. It is not clear how much of the improved consent rate resulted from the delivery of this information about the benefits of donation, nor is it clear what the attitudes of members of the community to the delivery of such information are. Therefore this randomised study was designed to compare attitudes of participants who watched one of two recorded family donation conversations where different information was provided. We hypothesised that participants who watched a donation conversation in which there was information provided about the benefits of donation (the “Supportive” conversation) would feel that the family had been adequately informed to make an enduring decision about donation, and would not feel that the doctor was being coercive, when compared to the group who watched a discussion without this positive information (the “Control” conversation).

## Materials and Methods

The Australian Red Cross Blood Service Human Research Ethics Committee approved this study. There were no patients involved, and as such no patient consent was required. Participants were given an overview of the requirements of their participation prior to following the link to the survey and could withdraw from the survey at any point; their subsequent voluntary involvement in the survey was taken as consent and the Australian Red Cross Blood Service Human Research Ethics Committee approved this consent procedure. The videos used, as made available in the links within this publication, were filmed using an actor in the role of patient, and the primary investigator in the role of doctor. Both have given their consent for the videos to be made available.

We placed a request for participation in our study in the electronic newsletter of an Australian commercial organisation with over 30,000 staff. Participants voluntarily responded by following a link to our survey. They were told that the study was to investigate the ways in which healthcare professionals communicate with the families of potential organ donors.

Participants were asked to answer questions pertaining to their demographics, their personal experience with organ and tissue donation and transplantation, and their own attitudes towards donation. They then read a short scenario [Box 1] prior to watching a video of a donation conversation. Participants were unaware that they had been randomly allocated to watch one of two videos. Both videos showed the same doctor talking to Joanne, an actor playing the role of a family member of a brain dead patient. Care was taken to ensure that the verbal and non-verbal communication style of the doctor, and all responses of Joanne, were identical in the two videos. They differed only in that the “Supportive” video contained information about the potential benefits of donation and transplantation, the rarity of the opportunity for donation, and the need for organs for transplantation, in addition to information about the organ donation process, in order to more fully support informed decision-making. The transcripts of the two videos, “Control” and “Supportive”, are shown in Boxes 2 and 3. The videos can be watched by following these links:

Control: http://youtu.be/86B51e5Rl-Y
Supportive: http://youtu.be/ewnIq8n6rW0

Participants were then asked to reflect on the video and assess their level of agreement with a number of statements, using a Likert scale. Four domains (using questions asked in random order) were used to assess the impact of the two different videos:

1. Uptake of factual information by study participants
  - Prior to watching the video, participants were asked if they agreed with the statement “most people are able to donate their organs when they die”.
  - After watching the video, they were then asked if they agreed with the statement “not many people die in a way that allows them to be an organ donor”.
2. Level of discomfort and general attitudes of the study participants
  - “I was uncomfortable listening to what the doctor was saying.”
  - “It is important that Joanne makes a decision that is right for her and for Shaun.”
3. Amount of information about organ donation provided by the doctor
  - “The doctor gave Joanne too much information about the benefits of organ donation.”
  - “The doctor provided enough information for Joanne to make a decision.”
  - “Joanne should have been given more information about the need for organ donation and the benefits of organ donation”
4. Influence and persuasion by the doctor
  - “The doctor cared about Shaun and his family.”
  - “The doctor was helping Joanne make a decision about organ donation that was best for Shaun and his family.”
  - “The doctor cared more about people waiting for transplants than about Shaun and his family.”
  - “The doctor was trying to convince Joanne to say yes to organ donation.”

**Box 1. Scenario**

Shaun is a young man who suffered a severe brain injury in a car accident 3 days ago and is in the Intensive Care Unit on a breathing machine (ventilator). A scan has shown that there is no blood flowing to his brain, a situation known as brain death. Brain death means that Shaun has died, even though a ventilator is supplying oxygen to the lungs and maintaining a heart beat.

Joanne is Shaun’s wife. Doctors have told her that Shaun has died. She has been spending time at his bedside in the Intensive Care Unit and is now meeting with the doctors to talk about what will happen next.

**Box 2. “Control” video transcript**

**DOCTOR**: Joanne, thank you for meeting with me again. I just wanted to start here by making sure that I had explained everything to you well the last time we met, and to see if you had any questions that you wanted to ask about Shaun’s death.

**JOANNE**: No, everything has been explained very well. I understand that Shaun has died.

**DOCTOR**: Joanne, I know this is a hard time to talk about this, but as part of our processes, and because Shaun died whilst on a ventilator, a breathing machine, I need to ask you about organ donation. I’m going to give you a bit of information about donation, and then I’m going to ask you to make a decision about whether or not Shaun would have wanted to donate.

Please feel free to interrupt me if you’d like to clarify anything that I’m saying, or to ask any questions as we go.

If you did decide to donate, Shaun would stay here in the Intensive Care Unit while we organise the donation process. The ventilator will continue, to make sure that oxygen is still being delivered to Shaun’s lungs, and his heart would continue to beat. We would do some blood tests to work out what Shaun might be able to donate, as well as some other tests, to make sure that we would get the best possible results from Shaun’s donation. That process can take as long as 24 hours, but often can be done more quickly than that and during that time, you’d be able to stay with Shaun for as long as you wanted to, at the bedside, and we’d be here to support you through the process. We’d keep you informed, as much as you want us to, of what’s happening.

We’re here to help you make the decision that is right for you, your family, and for Shaun. Do you think that organ donation is something that Shaun would have wanted?

**Box 3. “Supportive” video transcript**

**DOCTOR**: Joanne, thank you for meeting with me again. I just wanted to start here by making sure that I had explained everything to you well the last time we met, and to see if you had any questions that you wanted to ask about Shaun’s death.

**JOANNE**: No, everything has been explained very well. I understand that Shaun has died.

**DOCTOR**: Joanne, because Shaun has died whilst on a breathing machine, he has a rare opportunity to help other people by being an organ donor. I say that this is a rare opportunity because very few people actually die in a way that allows them to be organ donors. For example in Australia last year, in the whole of Australia, there were less than 400 people that were able to be organ donors. And this means that there are many people in Australia waiting for organ transplants.

So I’d like to give you some more information about donation to help you make the right decision for you and for Shaun, not just now, but into the future. A decision that you’ll be comfortable with in many years time.

I would like you to stop me if there is anything that you would like to clarify, and ask any questions that you would like as we go.

So, if Shaun was to be an organ donor, he could save or improve the lives of many people. For example, two people may be able to come off dialysis if they receive a kidney transplant, and someone who is waiting for a heart or lung transplant could have their life saved by Shaun’s donation.

If you did decide to donate, Shaun would stay here in the Intensive Care Unit while we organise the donation process. The ventilator will continue, to make sure that oxygen is still being delivered to Shaun’s lungs, and his heart would continue to beat. We would do some blood tests to work out what Shaun might be able to donate, as well as some other tests, to make sure that we would get the best possible results from Shaun’s donation. That process can take as long as 24 hours, but often can be done more quickly than that and during that time, you’d be able to stay with Shaun for as long as you wanted to, at the bedside, and we’d be here to support you through the process. We’d keep you informed, as much as you want us to, of what’s happening.

Joanne, some families have told us that for them, organ donation has been the one positive thing that they have been able to take away from what has otherwise been a really terrible experience. We’re here to help you make the decision that is right for you, for your family and for Shaun.

Tell me, what thoughts are you having at the moment about organ donation?

Statements were assessed by participants on a five point scale: strongly agree, agree, undecided, disagree or strongly disagree. Participants, who were unaware that they had been allocated to one of two randomised videos, submitted results electronically and anonymously after completing the survey.

Statistical analysis was undertaken in a blinded fashion, without knowledge of which video had been allocated to each group. All analysis was performed using SAS version 9.4 (SAS Institute Inc., Cary, NC, USA). Categorical values are reported as the number (proportion) and compared using chi-square tests for equal proportion. Normally distributed data were compared using student t-tests with results reported as mean (± standard deviation). Responses to ordinal questions were compared using Wilcoxon rank sum tests. To ensure observed results were not due to baseline imbalances, multivariable ordinal logistic regression was employed with ordinal responses treated firstly as five category outcomes with proportionality assumptions confirmed using a score test and goodness of fit determined using Pearson goodness of fit and deviance statistics. Additional sensitivity analysis was also performed with ordinal outcomes collapsed into 3 levels (agree, undecided & disagree). To account for multiple testing and to increase the robustness of findings, a two sided p-value of 0.01 was considered to be statistically significant.

With 252 participants per group, this study has an 80% power to detect a difference in score equal to 25% of 1 standard deviation with a 2-sided p-value of 0.05. To account for non-normality, this number was inflated by 15% in accordance with Lehmann[15].

## Results

**Fig 1:**
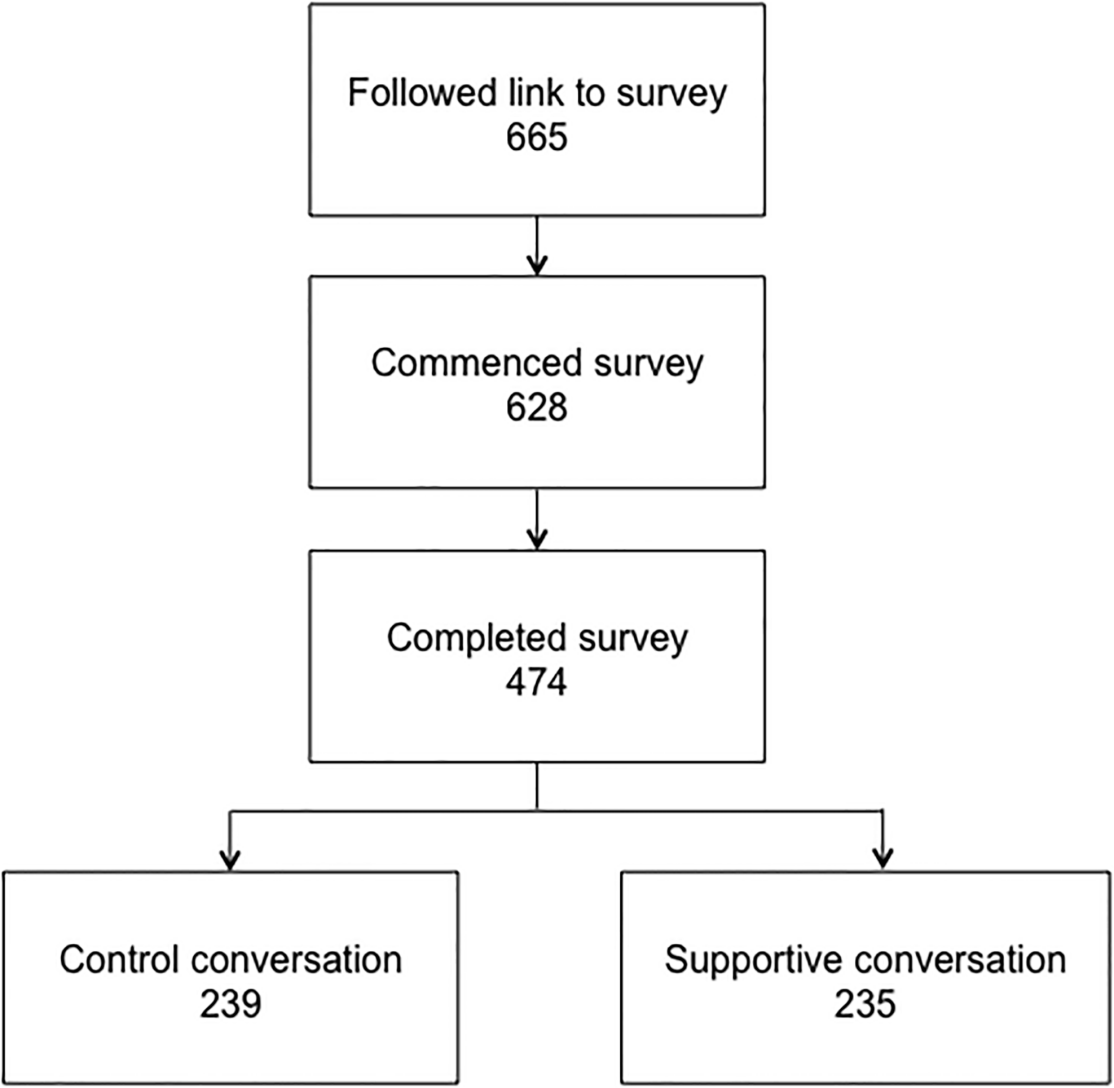
Consort diagram of participation in the survey.

665 people followed the link to the survey and entered demographic information. Of these, 628 commenced the survey and entered baseline information about opinions and experiences of organ donation and transplantation. The study cohort comprised 474 individuals who completed the survey, of whom 239 participants watched the “Control” video, and 235 the “Supportive” video (Fig 1). Comparisons between those who completed the survey and those who started but did not complete the survey questions showed that those who started but did not complete the survey were younger, more likely to be female, more often in the “other religion” group and more commonly knew someone who had been an organ or tissue donor. These results are shown in S1 Table.

### Baseline comparisons of “Control” and “Supportive” video groups

The two groups were well matched in most areas (Table 1). Those who watched the “Supportive” video less commonly spoke a language other than English at home and were slightly older. The commonest languages other than English spoken by participants were Greek (5 randomised to Control video, 7 to Supportive video), Cantonese (3, 3), Mandarin (2, 2), and Italian (1,3). Participants were equally likely to be registered on the Australian Organ Donor Register, to ever have been asked to consider organ or tissue donation, to know anyone who has been a donor or has received an organ or tissue transplant and were equally likely to consider organ donation themselves.

**Table 1:** Baseline demographics and comparisons between the two groups.

	Supportive (n = 235)	Control (n = 239)	P value
Age; mean (SD)	44.3 (11.6)	41.6 (10.7)	0.028
Male	132 (56.1%)	120 (50.2%)	0.19
Religion			
None	101 (43%)	94 (39.3%)	
Christian	113 (48.1%)	118 (49.4%)	0.55
Other	21 (8.9%)	27 (11.3%)	
Do you speak any language other than English at home?			
	26 (11.1%)	47 (19.7%)	0.009
Are you registered on the Australian Organ Donor Register?			
	102 (43.4%)	108 (45.2%)	0.70
Have you ever been asked to consider organ and tissue donation on behalf of a friend or relative?			
	35 (14.9%)	30 (12.6%)	0.46
Do you know anyone who has been an organ or tissue donor?			
	53 (22.6%)	44 (18.4%)	0.26
Do you know anyone who has received a human organ or tissue transplant?			
	71 (30.2%)	62 (25.9%)	0.30
Have you spoken to your family or friends about organ and tissue donation?			
	160 (68.1%)	163 (68.2%)	0.98
If you were ever in the position, would you wish to be an organ donor?			
Yes	174 (74%)	181 (75.7)	
No	6 (2.5%)	8 (3.3%)	0.73
Unsure	55 (23.4%)	50 (20.9%)	

Values represent number (%) who answered “Yes” to each question, unless stated

### Uptake of factual information by study participants

Uptake of factual information about organ donation was greater in the “Supportive” group (Table 2). Before watching the videos, there was no difference in knowledge about the rarity of the donation opportunity. Afterwards, the “Supportive” group were more likely to correctly report that not many people die in a way that allows them to be an organ donor. This remained significant even after adjusting for baseline imbalances between the two groups (adjusted odds ratio 7.57, 95% CI 5.19–11.04, p < 0.001).

**Table 2:** Uptake of factual information by study participants.

	Supportive (n = 235)	Control (n = 239)	P value
Pre-video: Most people are able to donate their organs when they die; n (%)			
Correct	131 (55.6)	134 (56.1)	
Undecided	56 (23.8)	72 (30.0)	0.06
Incorrect	48 (20.6)	33 (14.0)	
Post-video: Not many people die in a way that allows them to be an organ donor: n (%)			
Correct	191 (81.3)	83 (34.7)	
Undecided	25 (10.6)	86 (36.0)	<0.001
Incorrect	19 (8.1)	70 (29.3)	

P values represent Wilcoxon Rank Sum test for 3 ordinal categories

### Level of discomfort and general attitudes of study participants

**Fig 2:**
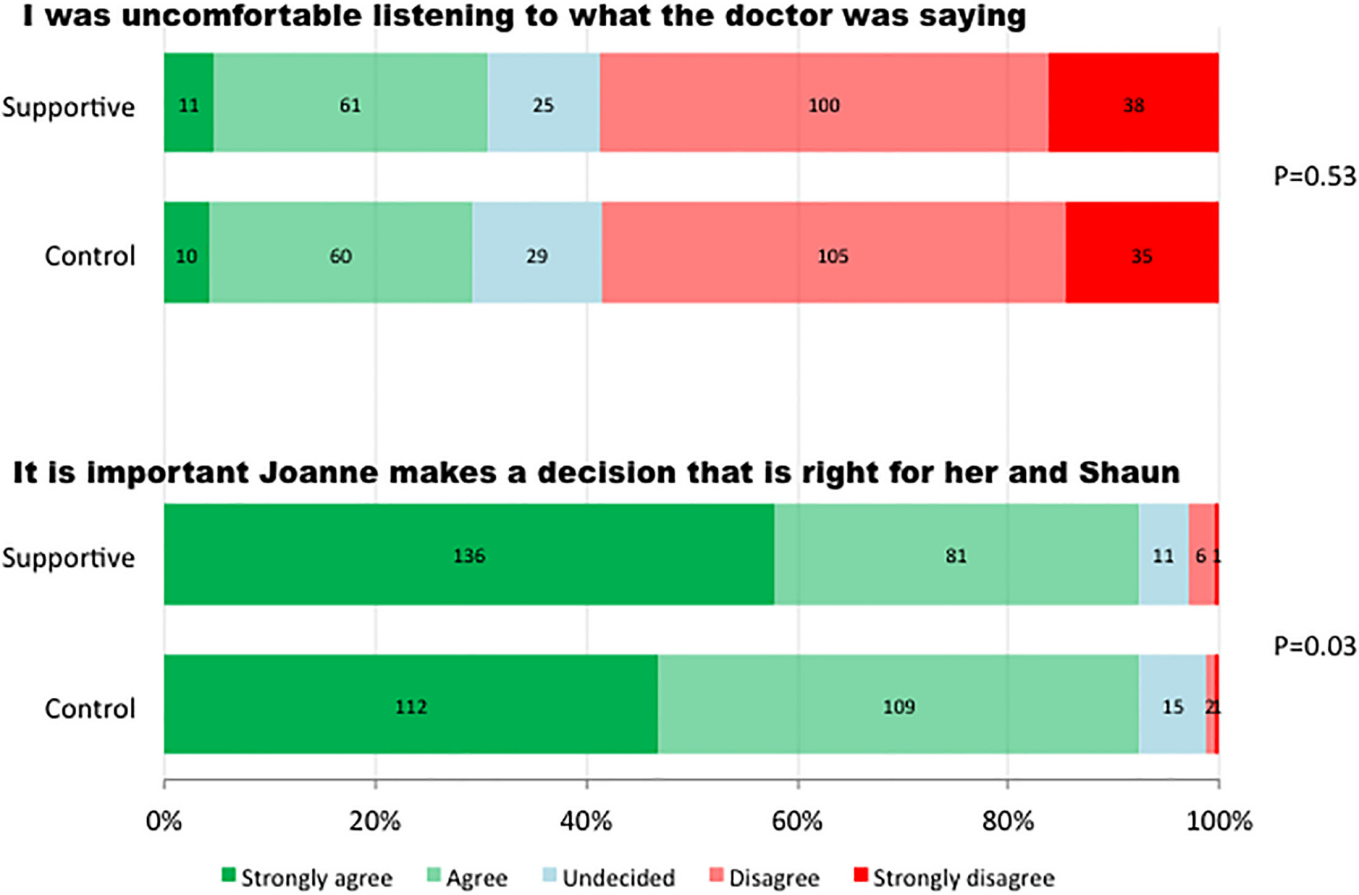
Level of discomfort and general attitudes of study participants.

The level of discomfort experienced by participants on watching the video was no different between the two groups (Table 3, Fig 2). There was a trend towards a greater proportion of participants who strongly agreed that it was “…important that Joanne makes a decision that is right for her and Shaun” in the group who watched the “Supportive” video. However this did not meet the pre-specified level for statistical significance either before or after adjusting for baseline differences between the groups.

**Table 3:** Univariable and multivariable ordinal logistic regression for progressive agreement in the “Supportive” video group compared to “Control” across 5 categories.

	Unadjusted Odds Ratio (95% CI)	P value	Adjusted* Odds Ratio (95% CI)	P value
**Level of discomfort and general attitudes of study participants**				
I was uncomfortable listening to what the doctor was saying	0.99 (0.71–1.37)	0.95	0.97 (0.70–1.35)	0.86
It is important that Joanne makes a decision that is right for her and Shaun	1.46 (1.03–2.08)	0.03	1.42 (1.00–2.04)	0.05
**Amount of information provided by the doctor**				
The doctor provided enough information for Joanne to make a decision	1.27 (0.91–1.77)	0.16	1.31 (0.93–1.83)	0.12
The doctor gave Joanne too much information about the benefits of organ donation	1.35 (0.94–1.93)	0.10	1.42 (0.99–2.05)	0.06
Joanne should have been given more information about the need for organ donation and the benefits of organ donation	0.34 (0.24–0.48)	<0.001	0.35 (0.25–0.50)	<0.001
**Influence and persuasion by the doctor**				
The doctor cared about Shaun and his family	1.24 (0.87–1.76)	0.24	1.26 (0.88–1.80)	0.21
The doctor was helping Joanne make a decision about organ donation that was best for Shaun and his family	1.11 (0.79–1.56)	0.53	1.15 (0.82–1.62)	0.43
The doctor cared more about people waiting for transplants than about Shaun and his family	1.25 (0.89–1.75)	0.20	1.34 (0.95–1.88)	0.10
The doctor was trying to convince Joanne to say yes to organ donation	3.00 (2.14–4.22)	<0.001	2.98 (2.11–4.20)	<0.001

* Adjusted for baseline imbalances: age and language spoken other than English

### Amount of information provided by the doctor

**Fig 3:**
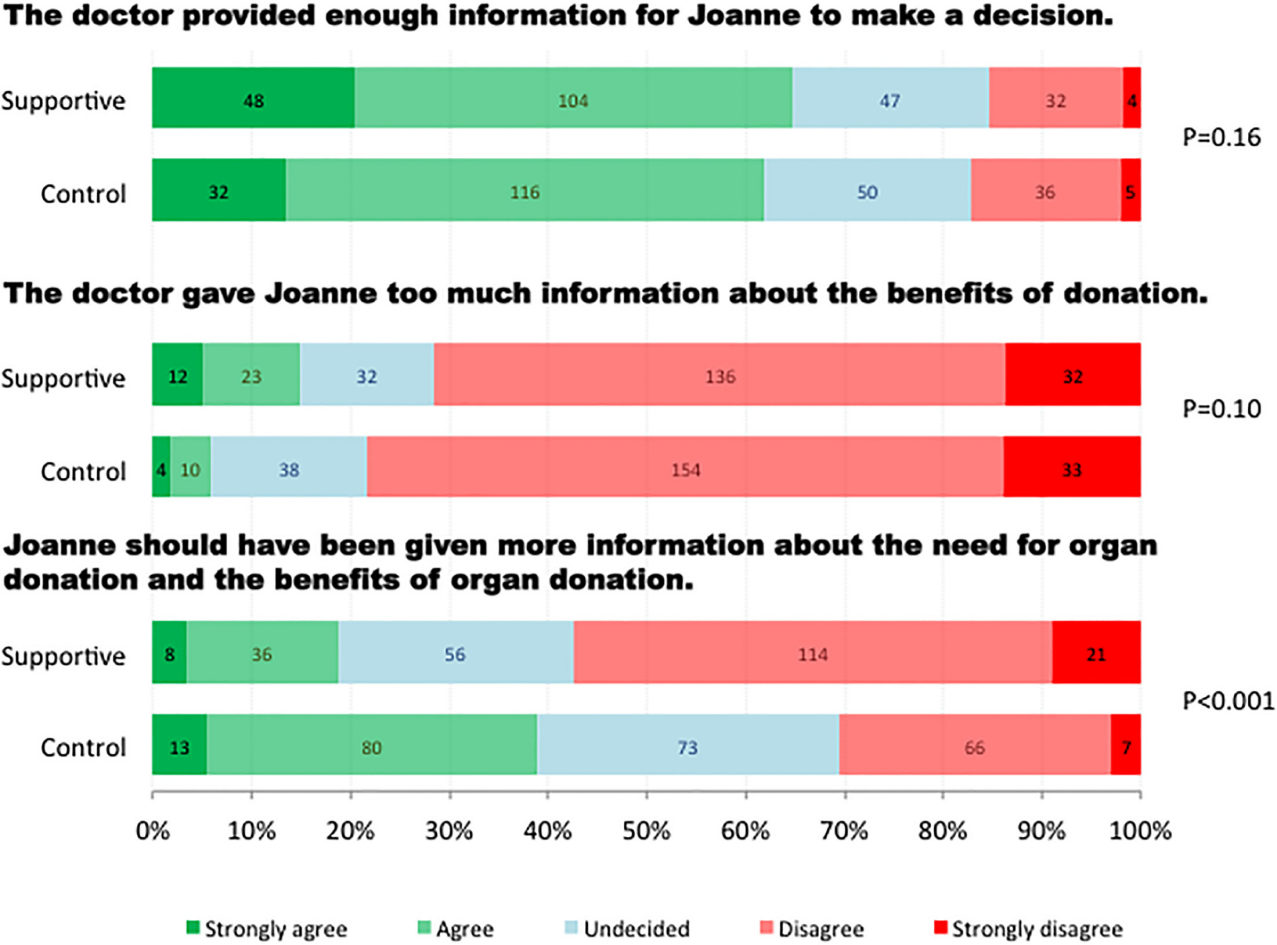
Amount of information provided by the doctor.

Participants who watched the “Supportive” conversation were less likely to report that “Joanne should have been given more information about the benefits of donation to help her make a decision” (Table 3 & Fig 3). This remained significant after adjusting for baseline differences in age and language spoken at home. Both groups were equally likely to agree that “the doctor provided enough information for Joanne to make a decision”, or to agree that “the doctor gave Joanne too much information”.

### Influence and persuasion by the doctor

**Fig 4:**
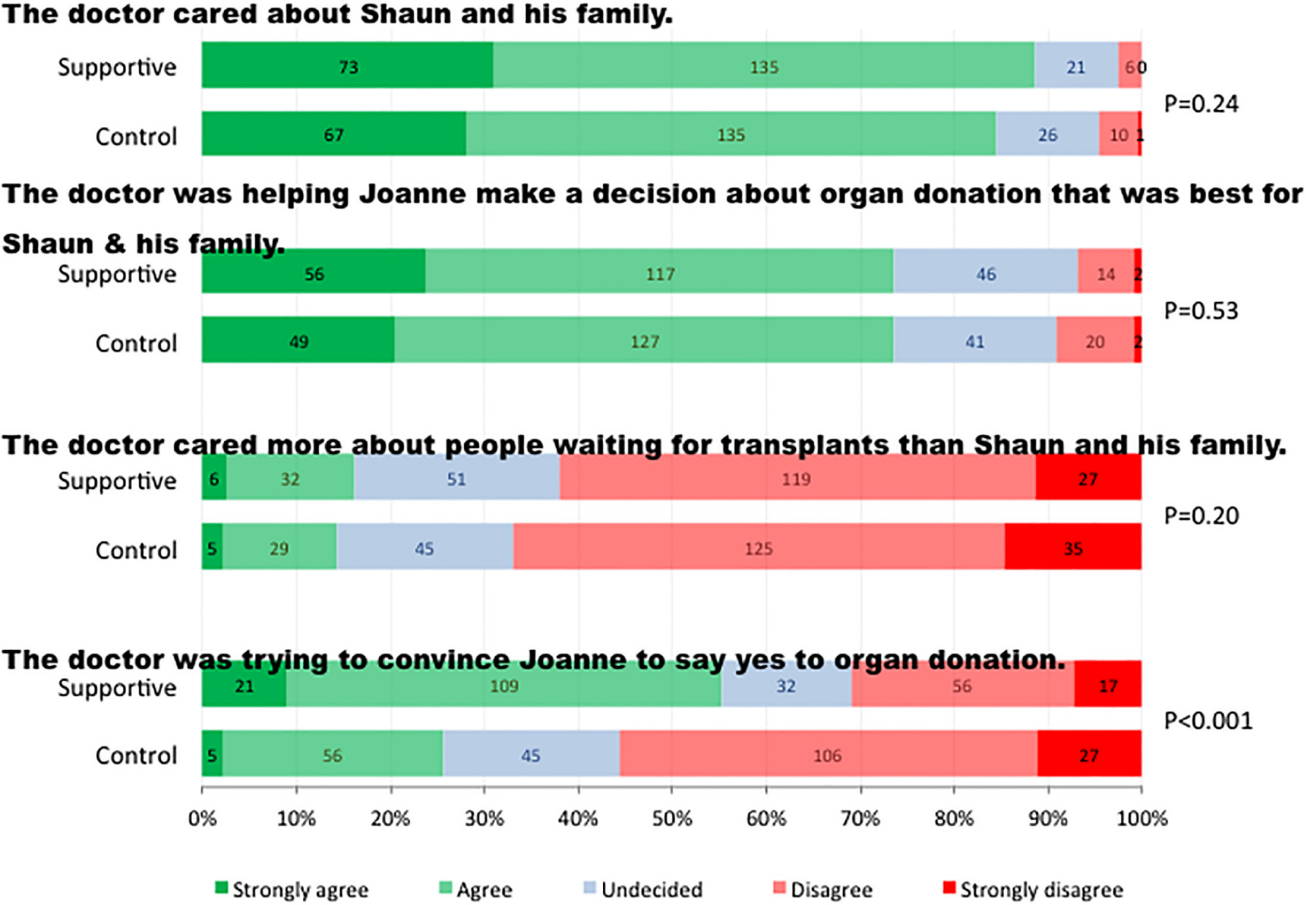
Influence and persuasion by the doctor.

The group who watched the “Supportive” video were more likely to agree that the doctor was trying to convince Joanne to say yes to organ donation (Table 3 & Fig 4). This remained significant after adjusting for baseline differences in age and language spoken at home. Both groups were equally likely to report that “the doctor cared about Shaun and his family”, that “the doctor was helping Joanne to make a decision about organ donation that was best for Shaun and his family”, and were no more likely to report that “the doctor cared more about transplant recipients than he did about Shaun and his family”.

## Discussion

We have shown that community members watching a video of a “Supportive” family donation conversation which contained positive information about the benefits of donation and transplantation, when compared to those watching a “Control” conversation, had greater uptake of factual information and were more likely to feel that the information provided to the family member was sufficient in quantity. They were more likely to report that the doctor was trying to convince the family member to say yes to donation. Despite this participants were no more likely to feel uncomfortable or to feel that the doctor cared more about transplant recipients than he did for the patient and their family. This suggests that community members are comfortable with health care staff providing more extensive information about donation and transplantation to family members, which may in turn be influential in consent for donation.

Prior to watching the videos, only half of the participants were able to correctly identify that organ donation is a rare opportunity. After watching the “Control” video, this fell to 35%, whereas after the “Supportive” video it increased to 81%. This suggests that participants remembered and were influenced by the facts provided in the “Supportive” video. It has been shown previously that knowing that the opportunity to be an organ donor is rare makes people more likely to want to become a donor^2^, and this information is therefore material to decision making about donation. We have shown that the rarity of the donation opportunity is not well known, and also that provision of this information in the video enhanced the participants’ understanding of this fact.

We found that people varied significantly in regards to what they felt to be the “right” amount of information to provide during a donation conversation. We have shown that the “Control” conversation was felt to provide inadequate information about the benefits of donation, and rarely felt to provide too much of this information. In those who watched the “Supportive” video, there were almost equal numbers of participants who reported that there was too much information, and not enough information, about the benefits of donation. This would suggest that the “Supportive” approach is closer to the right amount of information for most participants.

Approximately a quarter of those allocated to watch the “Control” video, which contained no specific positive information about organ donation, felt that the doctor was trying to convince Joanne to say yes to donation, compared to approximately half the respondents in the “Supportive” group. Despite this, very few respondents in either group felt that the doctor didn’t care about Shaun or that he was advocating for transplant recipients ahead of Shaun and his family. This supports the notion that donation requestors can be advocates for organ donation without affecting the sense of support felt by potential donor families, and without those family members feeling uncomfortable.

### Strengths and Limitations

We believe that this is the first prospective, randomised, blinded study assessing community members’ perceptions of family donation conversations. It is also the first study assessing the impact of information provision during a donation conversation in the Australian context.

We approached a large commercial organisation to assist in this survey because their workforce is extensive and diverse with respect to role, highest level of education attained, age, gender and cultural background. Although they were thought to be reflective of the broader community, generalisability to the whole Australian and wider international population cannot be directly assessed. The potential impact on the study findings of those who started but did not complete the survey is also unknown.

The “Control” group were slightly younger and contained a higher proportion that spoke a language other than English at home. However, by adjusting for these baseline differences using multivariable ordinal logistic regression, it appeared to be the video assignment that independently accounted for differences in the answers to questions, rather than baseline differences in demographics.

We assessed responses of community members who were not in the stressful position of actual potential donor families. It is not clear to what extent the situational challenges might have affected interpretation of these conversations. In addition, the ‘correct’ amount of information required will vary from one family to the next. It is important that health care staff tailor the amount and rate of information to the needs of each individual family member, which cannot be replicated in a study of this nature. Information about organ donation is provided for families over the course of several family meetings, and often over many hours. As such, participants in this survey were seeing only a very small part of this process. The proportion of respondents who reported that they were registered on the Australian Organ Donor Register was significantly higher than the community registration rate at the time of completing our survey (44% vs 23%)[16]. This may reflect a bias inherent in the voluntary participation nature of our survey. However, we found that support for organ and tissue donation was consistent with previous reports^2^. Further to this, since there was no difference in registration rate between the “Supportive” and “Control” groups, it is unlikely that this bias impacted on the comparison between the two groups.

We did not collect data regarding the highest level of education attained by study participants. This may have affected, in particular, the ability of study participants to learn and recollect new information about donation as assessed in regards to the rarity of donation. Whilst hopefully the randomisation process would have eliminated differences between the groups, we are not able to exclude a baseline imbalance in regards to participants’ level of education.

The two videos used in this study were closely matched in regards to the language used by the doctor discussing donation. However, they differed in that the Control video concluded with a closed question; “Do you think that organ donation is something that Shaun would have wanted?”. The Supportive video concluded with an open question; “What thoughts are you having at the moment about organ donation?”. This slight difference in script, whilst not specifically related to the benefits of organ donation and transplantation, may have affected the responses received from study participants.

Within Australia, the use of a trained requestor, as has recently been advocated^14^, will mean that the person raising donation will not always be a doctor and will not necessarily be known to the family. Although the videos in our study show a discussion lead by a doctor, we believe our findings are likely to be applicable to any individual requesting consent for donation and indeed to all clinical staff who provide information to family members of potential donors.

## Conclusion

This study supports the provision of information about the rarity of the donation opportunity and the benefits to others of organ donation during family donation conversations. Health care staff who discuss donation can present information about the donation process and the benefits of organ donation and transplantation, without risk of pressuring families into giving consent against the wishes of their loved one. It is, to our knowledge, the first prospective, randomised study of organ donation conversations, and provides important information to guide clinicians who discuss organ and tissue donation with families of potential donors.

## Supporting Information

S1 File All survey responses datasheet. (XLS)

S2 File Regression analysis data. (XLSX)

S1 Table Comparison between those who completed the survey and those who commenced but did not complete the survey. (DOCX)

S2 Table Level of discomfort and general attitudes of study participants (5 ordinal response categories collapsed into 3: agree, undecided or disagree). (DOCX)

S3 Table Amount of information provided by the doctor (5 ordinal response categories collapsed into 3: agree, undecided or disagree). (DOCX)

S4 Table Influence and persuasion by the doctor (5 ordinal response categories collapsed into 3: agree, undecided or disagree). (DOCX)
