# Supplementary material for: Randomised, Double Blind, Controlled Trial of the Provision of Information about the Benefits of Organ Donation during a Family Donation Conversation
Source: PLoS One. 2016 Jun 20;11(6):e0155778. doi: 10.1371/journal.pone.0155778 (PMC4913899; doi:10.1371/journal.pone.0155778)
Supplement: S1 Table — (DOCX) [file pone.0155778.s003.docx]

**S1 Table: Comparison between those who completed the survey and those who commenced but did not complete the survey**

|  |  | **Opened survey but dropped out**  **(n= 191)** | **Completed Survey**  **(n = 474)** | **P value** |
| --- | --- | --- | --- | --- |
| Age; mean (SD) |  | 40.0 (11.9) | 42.9 (11.9) | 0.003 |
| Male |  | 72 (38%) | 252 (53%) | <0.001 |
| Religion | None | 60 (31%) | 197 (42%) |  |
|  | Christian | 77 (40%) | 231 (49%) | <0.001 |
|  | Other | 54 (28%) | 46 (10%) |  |
| Do you speak any language other than English at home? | | | | |
|  |  | 35/154 (23%) | 73 (15%) | 0.036 |
| Are you registered on the Australian Organ Donor Register? | | | | |
|  |  | 67/154 (44%) | 210 (44%) | 0.86 |
| Have you ever been asked to consider organ and tissue donation on behalf of a friend or relative? | | | | |
|  |  | 20/154 (13%) | 65 (14%) | 0.82 |
| Do you know anyone who has been an organ or tissue donor? | | | | |
|  |  | 47/154 (31%) | 97 (20%) | 0.01 |
| Do you know anyone who has received a human organ or tissue transplant? | | | | |
|  |  | 49/154 (32%) | 133 (28%) | 0.37 |
| Have you spoken to your family or friends about organ and tissue donation? | | | | |
|  |  | 97/154 (63%) | 323 (68%) | 0.24 |
| If you were ever in the position, would you wish to be an organ donor? |  |  |  |  |
|  | Yes | 111/154 (72%) | 355 (75%) |  |
|  | No | 6/154 (4%) | 14 (3%) | 0.63 |
|  | Unsure | 37/154 (24%) | 105 (22%) |  |
| Values represent number (%) who answered “Yes” to each question, unless stated | | | | |
