# Supplementary material for: Randomised, Double Blind, Controlled Trial of the Provision of Information about the Benefits of Organ Donation during a Family Donation Conversation
Source: PLoS One. 2016 Jun 20;11(6):e0155778. doi: 10.1371/journal.pone.0155778 (PMC4913899; doi:10.1371/journal.pone.0155778)
Supplement: S2 Table — (DOCX) [file pone.0155778.s004.docx]

**S2 Table - Level of discomfort and general attitudes of study participants (5 ordinal response categories collapsed into 3: agree, undecided or disagree)**

|  |  | **Supportive**  **(n = 235)** | **Control**  **(n= 239)** | **Odds Ratio** | **P value** | ***Adjusted Odds Ratio** | ***Adjusted P value** |
| --- | --- | --- | --- | --- | --- | --- | --- |
| Post-video: I was uncomfortable listening to what the doctor was saying | | | | |  |  |  |
|  | Agree | 72 (30.6) | 70 (29.3) |  |  |  |  |
|  | Undecided | 25 (10.6) | 29 (12.1) | 1.02 (0.71-1.45) | 0.92 | 1.00 (0.69-1.43) | 0.98 |
|  | Disagree | 138 (58.7) | 138 (57.7) |  |  |  |  |
| Post-video: It is important that Joanne makes a decision that is right for her and Shaun | | | | |  |  |  |
|  | Agree | 217 (92.3) | 221 (92.5) |  |  |  |  |
|  | Undecided | 11(4.6) | 15 (6.3) | 0.96 (0.49-1.9) | 0.92 | 0.93 (0.46-1.86) | 0.84 |
|  | Disagree | 7 (3.0) | 3 (1.3) |  |  |  |  |
| Ordinal logistic regression for progressive agreement in the “Supportive” video group compared to “Control” across 3 categories  *Adjusted for baseline imbalances: age and language spoken other than English | | | | | | | |
