# Supplementary material for: Randomised, Double Blind, Controlled Trial of the Provision of Information about the Benefits of Organ Donation during a Family Donation Conversation
Source: PLoS One. 2016 Jun 20;11(6):e0155778. doi: 10.1371/journal.pone.0155778 (PMC4913899; doi:10.1371/journal.pone.0155778)
Supplement: S3 Table — (DOCX) [file pone.0155778.s005.docx]

**S3 Table: Amount of information provided by the doctor (5 ordinal response categories collapsed into 3: agree, undecided or disagree)**

| **Question** |  | **Supportive**  **(n = 235)** | **Control**  **(n= 239)** | **Odds Ratio** | **P value** | ***Adjusted**  **Odds Ratio** | **Adjusted**  **P value** |
| --- | --- | --- | --- | --- | --- | --- | --- |
| The doctor provided enough information for Joanne to make a decision | | | | |  |  |  |
|  | Agree or strongly agree | 152 (64.6) | 148 (61.9) |  |  |  |  |
|  | Undecided | 47 (20.0) | 50 (20.9) | 1.13 (0.78-1.63) | 0.52 | 1.16 (0.8-1.68) | 0.43 |
|  | Disagree or strongly disagree | 36 (15.3) | 41 (17.2) |  |  |  |  |
| The doctor gave Joanne too much information about the benefits of organ donation | | | | |  |  |  |
|  | Agree or strongly agree | 35 (14.9) | 14 (5.6) |  |  |  |  |
|  | Undecided | 32 (13.6) | 38 (15.9) | 1.55 (1.02-2.34) | 0.04 | 1.68 (1.1-2.57) | 0.02 |
|  | Disagree or strongly disagree | 168 (71.5) | 187 (78.2) |  |  |  |  |
| Joanne should have been given more information about the need for organ donation and the benefits of organ donation | | | | | | | |
|  | Agree or strongly agree | 44 (18.7) | 93 (38.9) |  |  |  |  |
|  | Undecided | 56 (23.8) | 73 (30.5) | 0.34 (0.24-0.48) | <0.001 | 0.35 (0.25-0.49) | <0.001 |
|  | Disagree or strongly disagree | 135 (57.4) | 43 (18.0) |  |  |  |  |
| Ordinal logistic regression for progressive agreement in the “Supportive” video group compared to “Control” across 3 categories  *Adjusted for baseline imbalances: age and language spoken other than English | | | | | | | |
