# Supplementary material for: Randomised, Double Blind, Controlled Trial of the Provision of Information about the Benefits of Organ Donation during a Family Donation Conversation
Source: PLoS One. 2016 Jun 20;11(6):e0155778. doi: 10.1371/journal.pone.0155778 (PMC4913899; doi:10.1371/journal.pone.0155778)
Supplement: S4 Table — (DOCX) [file pone.0155778.s006.docx]

**S4 Table: Influence and persuasion by the doctor (5 ordinal response categories collapsed into 3: agree, undecided or disagree)**

| **Question** |  | **Supportive**  **(n = 235)** | **Control**  **(n= 239)** | **Odds Ratio** | **P value** | ***Adjusted**  **Odds Ratio** | **Adjusted**  **P value** |
| --- | --- | --- | --- | --- | --- | --- | --- |
| The doctor cared about Shaun and his family | | | | |  |  |  |
|  | Agree or strongly agree | 208 (88.5) | 202 (84.5) |  |  |  |  |
|  | Undecided | 21 (8.9) | 26 (10.9) | 1.43 (0.84-2.43) | 0.19 | 1.49 (0.86-2.56) | 0.15 |
|  | Disagree or strongly disagree | 6 (2.6) | 11 (4.6) |  |  |  |  |
| The doctor was helping Joanne to make a decision about organ donation that was best for Shaun and his family | | | | |  |  |  |
|  | Agree or strongly agree | 173 (73.6) | 176 (73.6) |  |  |  |  |
|  | Undecided | 46 (19.6) | 41 (17.2) | 1.03 (0.69-1.55) | 0.88 | 1.05 (0.7-1.58) | 0.81 |
|  | Disagree or strongly disagree | 16 (6.8) | 22 (9.2) |  |  |  |  |
| The doctor cared more about people waiting for transplants than about Shaun and his family | | | | |  |  |  |
|  | Agree or strongly agree | 38 (16.2) | 34 (14.2) |  |  |  |  |
|  | Undecided | 51 (21.7) | 45 (18.8) | 1.22 (0.84-1.76) | 0.29 | 1.36 (0.93-1.98) | 0.11 |
|  | Disagree or strongly disagree | 146 (62.1) | 160 (66.9) |  |  |  |  |
| The doctor was trying to convince Joanne to say yes to organ donation | | | | |  |  |  |
|  | Agree or strongly agree | 130 (55.3) | 61 (25.5) |  |  |  |  |
|  | Undecided | 32 (13.6) | 45 (18.8) | 3.15 (2.22-4.48) | <0.001 | 3.15 (2.2-4.49) | <0.001 |
|  | Disagree or strongly disagree | 73 (31.1) | 133 (55.6) |  |  |  |  |
| Ordinal logistic regression for progressive agreement in the “Supportive” video group compared to “Control” across 3 categories  *Adjusted for baseline imbalances: age and language spoken other than English | | | | | | | |
